# Structural diversity of marine anti-freezing proteins, properties and potential applications: a review

**DOI:** 10.1186/s40643-022-00494-7

**Published:** 2022-01-22

**Authors:** Soudabeh Ghalamara, Sara Silva, Carla Brazinha, Manuela Pintado

**Affiliations:** 1 Universidade Católica Portuguesa, CBQF – Centro de Biotecnologia e Química Fina – Laboratório Associado, Escola Superior de Biotecnologia, Rua Diogo Botelho 1327, 4169-005 Porto, Portugal; 2 LAQV/Requimte, Faculdade de Ciências E Tecnologia, Universidade Nova de Lisboa, Campus de Caparica, 2829-516 Caparica, Portugal

**Keywords:** Marine antifreeze proteins, Ice recrystallization inhibition (IRI), Thermal hysteresis (TH), Function, Potential applications

## Abstract

Cold-adapted organisms, such as fishes, insects, plants and bacteria produce a group of proteins known as antifreeze proteins (AFPs). The specific functions of AFPs, including thermal hysteresis (TH), ice recrystallization inhibition (IRI), dynamic ice shaping (DIS) and interaction with membranes, attracted significant interest for their incorporation into commercial products. AFPs represent their effects by lowering the water freezing point as well as preventing the growth of ice crystals and recrystallization during frozen storage. The potential of AFPs to modify ice growth results in ice crystal stabilizing over a defined temperature range and inhibiting ice recrystallization, which could minimize drip loss during thawing, improve the quality and increase the shelf-life of frozen products. Most cryopreservation studies using marine-derived AFPs have shown that the addition of AFPs can increase post-thaw viability. Nevertheless, the reduced availability of bulk proteins and the need of biotechnological techniques for industrial production, limit the possible usage in foods. Despite all these drawbacks, relatively small concentrations are enough to show activity, which suggests AFPs as potential food additives in the future. The present work aims to review the results of numerous investigations on marine-derived AFPs and discuss their structure, function, physicochemical properties, purification and potential applications.

**Graphical Abstract:**

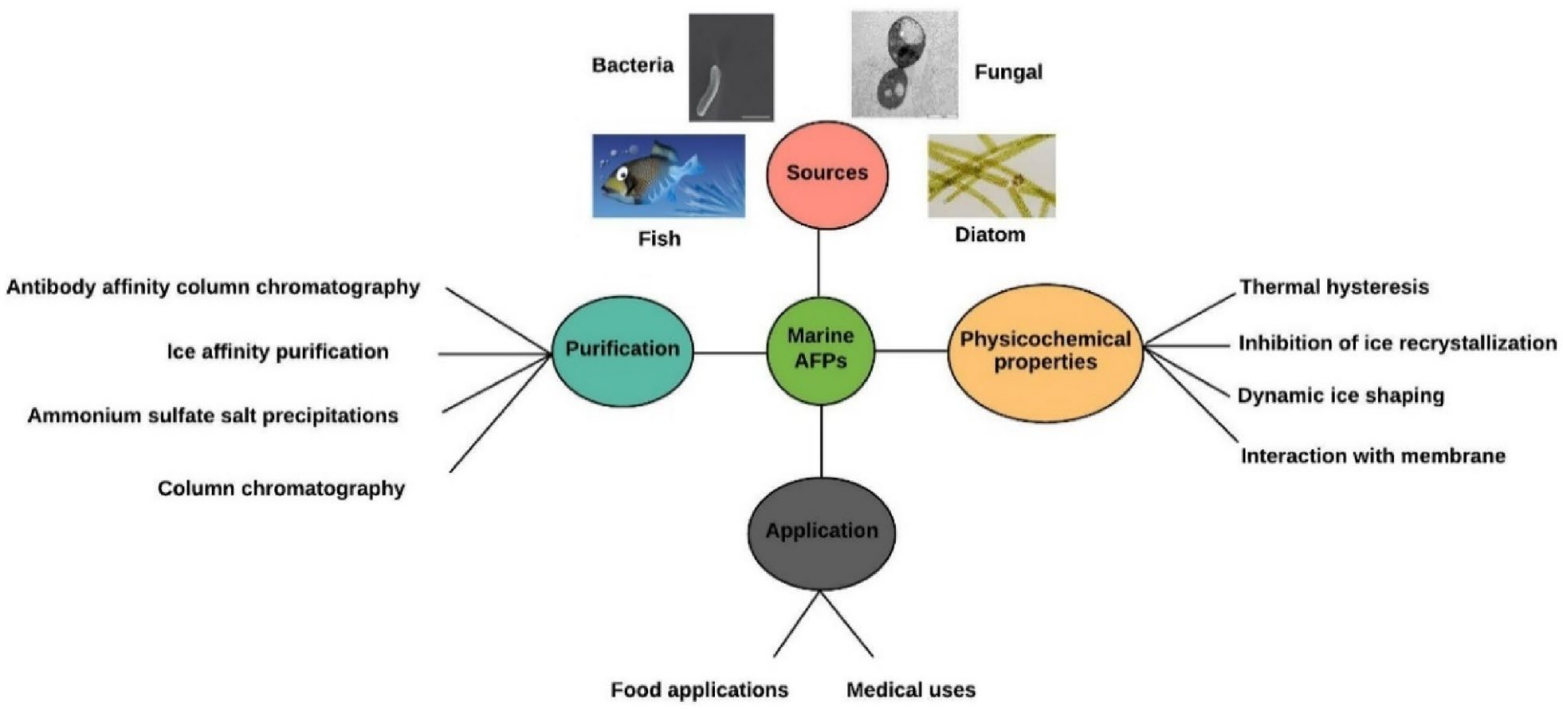

## Introduction

Cold habitats are crucial to the planet with temperatures of about 85% of the earth being (permanent or seasonal) below 5 °C (from the deep sea to the alpine and the Antarctic to the Arctic) (Hassan et al. 2016). Moreover, nearly two-thirds of the earth's surface are underwater with average surface temperatures ranging from − 2 to 30 °C based on the latitude. Seawater temperatures in the polar regions are persistently sub-zero, temperatures which can cause extreme damage, if not deadly, to polar fish. As a cold survival mechanism, various polar fish develop anti-freezing proteins/peptides (AFPs) or antifreeze glycol proteins/peptides (AFGPs) to protect themselves against freezing damage. Devries and Wohlschlag (1969) first detected such proteins in the blood of fishes residing in regions, where the sea freezes and they reduce the freezing point of the fish's blood to below the seawater freezing point, with no significant increase in the osmotic pressure of the plasma. The term ice structuring proteins (ISPs) has been suggested as an appropriate name, since AFPs bind to and influence ice crystal growth (Crevel et al. 2002).

AFPs are a series of proteins that bind to ice crystal planes in a structured mode, depending on the type of proteins inhibiting the formation of ice crystals (within a specific temperature range). Adding non-volatile solutes to pure water decreases the solution's equilibrium freezing point linearly in a concentration-dependent manner, by 1.858 °C per 1000 mol of osmotically active solutes per kg of water (Osm) of dissolved solute particles. This is one of the solutions' colligative properties and as a consequence of Raoult's law regulating the vapor pressure of the solution. AFPs occur in significant physiological concentrations (mass/volume), but because of their macromolecular nature, these turn into very low osmolar concentrations. For example, the Antarctic eelpout *P. brachycephalum* Type III AFP of about 7000 Da circulates in the blood at about 20 mg/ml, but this corresponds to an osmotic concentration of just 3 mOsm and can only affect 0.0056 °C of freezing point depression equilibrium based on colligative relationships (DeVries and Cheng 2005). However, where the solution is comprised of small ice crystals, upon cooling, AFPs prevent the growth of crystals until a certain temperature is achieved, after which the ice crystals dramatically expand. This temperature level is called the hysteresis freezing point (HFP), and the difference between the solutions’ melting point and HFP being called thermal hysteresis (TH) or anti-freezing activity. The temperature interval between the solutions’ melting point and the HFP is known as hysteresis gap (Wathen and Jia 2005).

AFPs exhibit considerable diversity in tertiary structures (Graether et al. 2000; Ko et al. 2003; Liou et al. 2000; Nishimiya et al. 2008; Pentelute et al. 2008; Sicheri and Yang 1995). This diversity is partly due to their independent evolutionary origins (Cheng 1998; Fletcher et al. 2001), and partly to the heterogeneity of their natural ligand, ice (Davies et al. 2002). Hexagonal ice poses a variety of different planes (expressed as Miller indices) of water molecules to which the AFP can develop an affinity. Although the specificity of various ice planes is the main determinant of anti-freezing activity (Scotter et al. 2006), the mechanism by which the AFP is associated with the ice remains unknown.

AFPs have been identified in a variety of cold-adapted marine organisms, such as fish (Hanada et al. 2014), bacteria (Do et al. 2014; Gilbert et al. 2005; Raymond et al. 2007; Singh et al. 2014), microalgae (Gwak et al. 2010; Janech et al. 2006; Jung et al. 2014; Kang and Raymond 2004; Krell et al. 2008; Raymond et al. 2009), and fungi (Boo et al. 2013; Hashim et al. 2013; Lee et al. 2010). AFPs inhibit ice creation by interacting with ice cascade gaps and preventing ice crystals from binding to each other (Nada and Furukawa 2012; Raymond and DeVries 1977). AFPs are also classified based on their TH (Garnham et al. 2008, 2011; Yang and Sharp 2004). There are two types of AFPs according to the TH: moderate AFPs and hyperactive AFPs. Moderate AFPs, present in Antarctic fish, have a TH of approximately 0.5–1 °C with an AFPs concentration level of 1 mg/ml, while hyperactive AFPs, such as those identified in mealworm *Tenebrio molitor*, could show a TH level up to 6 °C, with a concentration of 1 mg/ml of AFPs (Graham et al. 1997). Moreover, hyperactive AFPs existing in Antarctic bacterium *Marinomonas primoryensis* could display a TH of 2 °C at 0.1 mg/ml (Garnham et al. 2008).

Moderate and hyperactive AFPs act both by reducing the water freezing point and as ice growth inhibitors and prevent recrystallization during storage. As such, their application in certain areas such as the food industry may be of interest. Particularly when trying to improve the properties of frozen foods, the freezing/thawing qualities of ice creams (Regand and Goff 2006) or foodstuffs (Li and Sun 2002). Furthermore, their use has resulted in an improvement of the half-life of frozen dough (Zhang et al. 2007), facilitated cryosurgery that relies on intracellular ice formation (Koushafar and Rubinsky 1997), and the cryopreservation of red blood cells (RBC), organs, tissues and membranes (Amir et al. 2003; Chao et al. 1996).

In this paper, we discuss the various types of AFPs found in marine species, such as fish, diatom, bacteria, and fungi, as well as their structures, physicochemical properties (TH, IRI, DIS, and interaction with membranes), purification (ice affinity and falling water ice affinity purification), action mechanisms (TH effect and adsorption–inhibition process) and possible applications in food and medical products.

## Marine AFPs

There’s a high and increasing demand for proteins and peptides, due to their nutritional properties, but also their health promoting properties. As such, endogenous marine proteins/peptides have demonstrated to be an interesting potential source of food ingredients and pharmaceutical agents. AFPs, produced by a wide range of marine species living in cold environments, such as fish, bacteria, fungi, microalgae, and diatoms, are among the most important types of marine proteins that can be exploited.

### Fish AFPs structure and amino acids composition

Marine Teleosts, taking polar waters as habitat, have a body temperature of around 1.9 °C which is the same as the temperature of the aquatic environment. However, their body fluids are hypo-osmotic to seawater, which has a melting point of ca. 0.7 °C (Gordon et al. 1962; Scholander et al. 1957).

**Fig. 1:**
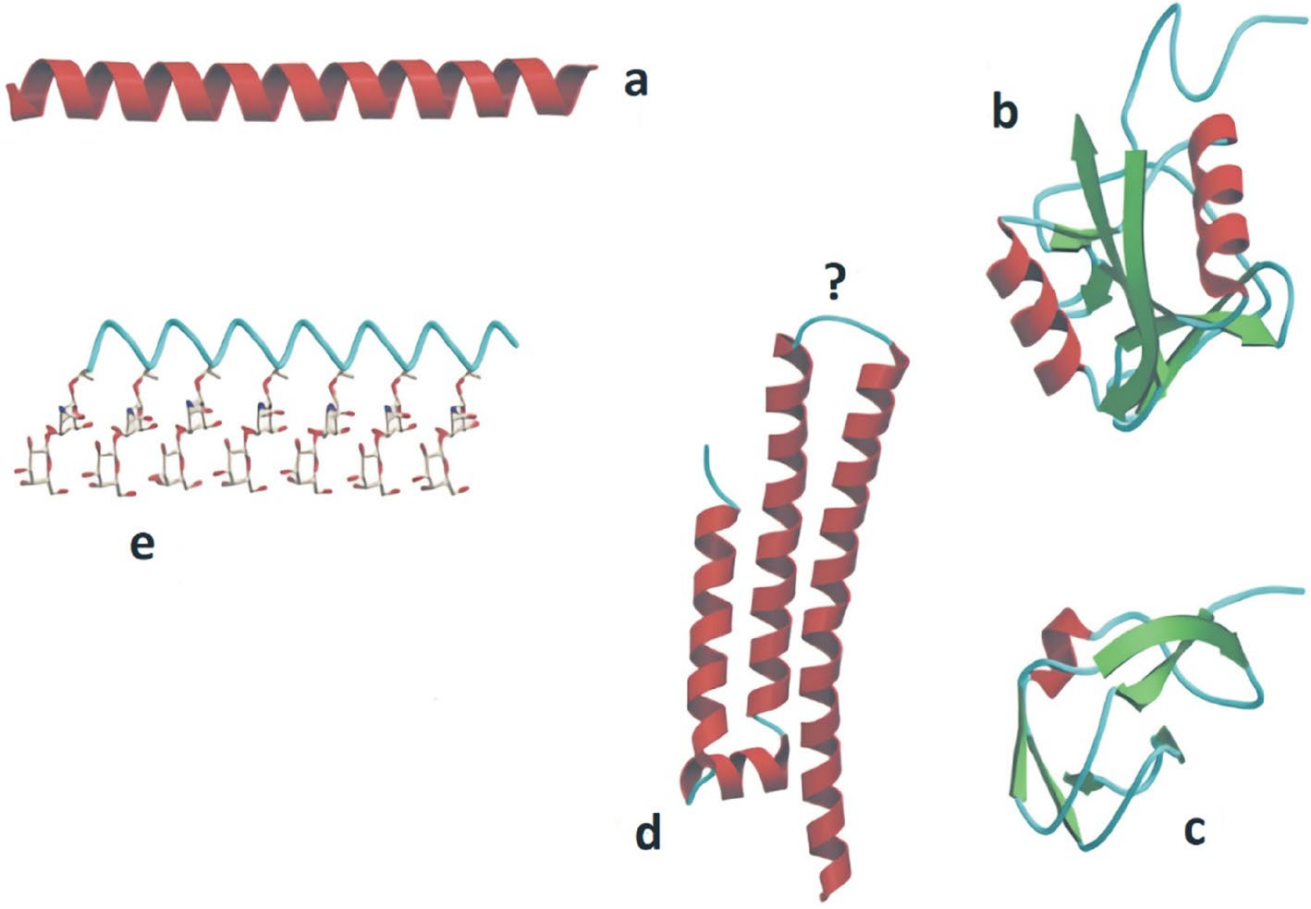
AFPs structures. Representative structures are depicted on a scale for the five types of fish AFP with α helices in red, β strands in green and coil in cyan. **a** AFP type I from winter flounder (1WFA). **b** AFP type II from sea raven (2AFP). **c** AFP type III from ocean pout (1MSI). **d** Type IV AFP designed as a helix bundle; the question mark showed uncertainty about its repetitive character. **e** AFGP described as an expanded left-handed helix with disaccharides (Yeh and Feeney 1996). Structures have been developed using Molscript (Kraulis 1991)

In the 1950s, experiments by Canadian scientist Schlender led him to believe that Arctic fish blood contained an anti-freezing ingredient (Scholander et al. 1957). The investigation from Antarctic fish enabled Arthur Devries to isolate AFPs (Yang et al. 1998). Such proteins were later referred to as AFGPs to distinguish them from non-glycoprotein biological antifreeze agents (Fletcher et al. 2001). In fish, a total of five structurally distinct AFPs have been identified so far and classified as AFPs type I, II, III, IV, and AFGPs based on their distinct structural features and physicochemical properties (Fletcher et al. 2001) (Fig. 1).

### AFPs

#### Type I AFPs

Type I AFPs are distinguished by a high alanine content (> 60%), a high helical content, and 11-residue repeat sequences that begin with threonine. This protein class was discovered in the blood serum of the winter flounder, *Pseudopleuronectes americanus*, which inhabits in close to shore waters off the coast of Nova Scotia (Duman and DeVries 1974, 1976). Since then, type I proteins have been discovered in the skins of the winter flounder (Gong et al. 1996), the yellowtail flounder, *Limanda ferruginea* (Scott et al. 1987), Alaskan plaice (Knight et al. 1991), the grubby sculpin, *Myoxocephalus aenaeus* and the Arctic sculpin (Chakrabartty et al. 1988).

Duman and DeVries (1974, 1976) identified two main proteins from the winter flounder. These are known as HPLC6 and HPLC8 in the literature (Hew et al. 1984).The sequences are nearly identical, with the exception of residues 22 and 26. HPLC6 is one of the few type I AFPs for which both a solid state structure (Sicheri and Yang 1995) and thorough nuclear magnetic resonance (NMR) studies have been described (Gronwald et al. 1996; Sönnichsen et al. 1998). Three 11-amino-acid repetitions of the sequence ThrX2AsxX7 are found in the 37-residue protein, where X is frequently alanine or another amino acid that promotes α-helix formation. HPLC6 has undergone extensive conformational studies utilizing circular dichroism (CD), NMR, and X-ray diffraction, revealing that it is fully α-helical in conformation, with the exception of the final unit, which adopts a 310-helix conformation.

The six type I proteins isolated from shorthorn (Hew et al. 1985; Fletcher et al. 1982), grubby (Chakrabartty et al. 1988), and Arctic sculpins (Yang et al. 1988), as well as SAFP1 from the winter flounder (Gong et al. 1996), are distinct from the other sequences in the composition of the N-terminal, while these proteins have the same 11-residue internal repeat structure as the other type I proteins, the repeat structure is less strict, with minor variations in the Asx residues and more lysine residues. Shorthorn sculpin proteins have two structural and presumably functional domains (Hew et al. 1985).

The 9-residue N-terminal region of domain SS8 was cleaved and known to contain little secondary structure. The second domain is helical, with two continuous 11-amino acid residue repeats, followed by another relatively similar repeat. SS8 has a high helical content (73%), while SS3 has a moderate helical content (about 45%) (Wierzbicki et al. 1996).

Models of how these proteins bind to ice were developed using the type I AFP structures and data from other biophysical research. The AFP binds to certain ice planes, which were disclosed by ice etching, a particularly powerful approach. The HPLC6 isoform has been demonstrated to bind to the (2 0–2 1) face along its < 0 1–1 2 > side, whereas the AFP from sculpin (*Myoxocephalus scorpius*) binds to the secondary prism planes (2–1–1 0) (Knight et al. 1991).

#### Type II AFPs

Type II AFPs are cysteine-rich proteins ranging in size from 11 to 24 kDa that are split into two categories based on the need for 1 mol of Ca^2+^ for TH and ice-shaping functions. Ca^2+^-dependent type II AFPs have been identified in Japanese smelt (*Hypomesus nipponensis*) (jsAFP) (Yamashita et al. 2003), rainbow smelt (*Osmerus mordax*) (smeltAFP), and Atlantic herring (*Clupea harengus*) (hAFP) (Ewart et al. 1990). Ca^2+^-independent AFPs have been found in the sea raven (*Hemitripterus americanus*) (srAFP) (Ng et al. 1986) and the longsnout poacher (*Brachyopsis rostratus*) (BrAFP) (Nishimiya et al. 2008).

Despite the fact that Ca^2+^-dependent and -independent species share just 40% of their sequences, an X-ray investigation revealed that they are similar to each other (Nishimiya et al. 2008) and to the Ca^2+^-dependent (C-type) lectin reference (Ewart et al. 1998). Although the exact location of the ice binding surface in jsAFP has yet to be determined, mutation studies have previously revealed that it is near the Ca^2+^-binding loop (Yasui et al. 2008). The ice-binding mechanism involves two Ca^2+^-coordinating residues (D94 and E99) as well as two polar residues (T96 and T98) with a protein-bound Ca^2+^ ion, according to docking simulations (Liu et al. 2007). The Ca^2+^-dependent and Ca^2+^-independent type II AFPs are thought to have different mechanisms for avoiding ice formation (Liu et al. 2007). This family of freeze-preventing proteins, which is assumed to have developed from the sugar-binding domain of C-type (Ca^2+^-dependent) lectins, likewise has 10 cysteine residues, resulting in five disulfide bridges in the same places (Liu et al. 2007; Graham et al. 2008; Drickamer et al. 1999; Ewart et al. 1992). The type II AFP has five disulfide bridges, whereas C-type lectins normally have two to three disulfide bridges. At 1.70 Å and 1.34 Å resolutions, the crystal structures of type II AFP from herring (*Clupea harengus*) with 127 residues (Liu et al. 2007) (2PY2) and longsnout poacher (*Brachyopsis rostratus*) (lpAFP) with 130 residues (Nishimiya et al. 2008) (2ZIB) have been discovered. The root-mean-square deviation of Cα atoms in the superimposed structures is 1.1 Å, and these two proteins have 39% sequence similarity. Ca^2+^ is required for hAFP to function, whereas lpAFP is not.

Two α-helices and eight β-strands constitute their total fold. Type II AFPs are distinguished by the presence of disulphide linkages. All 10 cysteines in lpAFP and hAFP are coupled to form five disulphide linkages. As a result, structurally, these two proteins have a lot in common. It appears to be quite remarkable that two proteins with considerable structural similarities may perform the same function in two distinct ways, one requiring Ca^2+^ and the other not.

#### Type III AFPs

Type III AFPs are not similar to any other AFPs, they are small globular proteins, with an average molecular weight of 6.5 kDa polymer, present in the ocean pout, Antarctic eelpout (*Macrozoarces americanus*) and wolf fish (Antson et al. 2001; Yeh and Feeney 1996), which have fewer alanine residues and no cysteine residues. AFP III molecules have no unique primary structure and no carbohydrates. Type III AFPs are homologous to the enzyme sialic acid synthase's (SAS) C-terminal domain (Baardsnes and Davies 2001) and probably evolved from it by gene duplication and divergence (Deng et al. 2010). SAS from an Antarctic eelpout, a fish producing type III AFP, has been found to have ice-binding activity and the evidence of gene duplication is apparent in the genome of the fish.

Fish AFPs III are globular proteins that consist of several short β-strands and a helical turn. Based on variations in their isoelectric points, type III AFPs have been split into two subsets: quaternary-amino-ethyl (QAE) and sulfopropyl (SP) sephadex-binding isoforms (Hew et al. 1988), and the QAE proteins may be further separated into QAE1 and QAE2 subgroups (Nishimiya er al. 2005). The Japanese notched-fin eelpout (*Zoarces elongates Kner*) generates 13 distinct isoforms of type III AFP (denoted nfeAFP1–nfeAFP13), which have been classified into six SP (nfeAFP1–nfeAFP6), four QAE1 (nfeAFP7–nfeAFP10), and three QAE2 (nfeAFP11–nfeAFP13) (Nishimiya et al. 2005). The ice-binding surface of a type III AFP is made up of solvent-exposed residues from two portions of the amino acid sequence (residues 9–21 and 41–44) (Deluca et al. 1998; Graether et al. 1999; Baardsnes et al. 2002; Garnham et al. 2010).

The ice-binding surface is split into two neighboring surfaces that attach to the ice crystal's pyramidal and primary prism planes, accordingly, and its relative hydrophobicity and flatness are regarded to be critical for antifreeze action (Garnham et al. 2012). The QAE1 isoform binds both the pyramidal and primary prism planes of ice crystals via these two ice-binding surfaces and is capable of stopping ice development (Garnham et al. 2012). However, the SP and QAE2 isoforms can only adhere to the pyramidal ice plane and so have little or no TH activity (Garnham et al. 2012). From the Antarctic eelpout (*Lycodichthys dearborni*), three main and at least five small variations of AFPIII have been identified (Wang et al. 1995a, b). The two primary AFPs (RD1, RD2) have a 7 kDa size, a unique 64-aa AFP domain, and are identical to AFPIII seen in other fishes. A third (RD3) isoform was discovered in 1995, comprising two 7 kDa AFP domains linked in tandem by a 9-residue linker sequence (-Asp-Gly-Thr-Thr-Ser-Pro-Gly-Leu-Lys-) (Wang et al. 1995a).

#### Type IV AFPs

*Myoxocephalus octodecimspinosis* was identified in longhorn sculpin plasma (LHS) as the first type IV AFP found in the coastal waters of Massachusetts and New Hampshire (Deng et al. 1997). This 12.3 kDa protein, originally referred to as LS-12, contained 108 amino acids and was relatively glycine rich (17%). The presence of a 20 amino acid N-terminal signal sequence was correlated with its export to the bloodstream. The sequence also showed its similarities to the other four-helix bundles of serum/hemolymph apolipoproteins, such as apolipoprotein E from the guinea pig, and apolipophorin III from the African locust (Deng et al. 1997). Subsequently, through CD analysis, Deng and Laursen (1998) defined its substantial α-helicity (60% at 1 °C) and developed a protein helix bundle model that was supported by partial proteolysis. Typically, thermolysin cleavage was pronounced in the helix-connecting loop regions and mutated within the helical regions. The left-hand model, antiparallel four-helix bundle had four amphipathic main helices paired with their hydrophobic surfaces forming the core of the bundle and their hydrophilic surfaces oriented outward to the solvent. Significantly, along with the common findings of AFP IV homologs, their abundant expression has recently been observed in oocytes (Breton et al. 2012; Goetz et al. 2006), and embryos (Liu et al. 2009) from some of the teleost fish. The genomic structure of AFP IV was only identified by Lee et al. (2011). Initially, two AFP IV homologs have been documented from databases of three-spined sticklebacks (*Gasterosteus aculeatus*) and Atlantic salmon (*Salmo salar*) (Lee et al. 2011). However, their distributions in the chromosomes have not been explained. Along with type IV, AFP has been found in temperate, subtropical and tropical fishes that do not need to prevent freezing (Gauthier et al. 2008; Lee et al. 2011; Liu et al. 2009), their biological functions have been hypothesized to bind to lipids or ligands other than ices because of the similarity of helix bundle to some apolipoproteins (Apos) (Breton et al. 2012; Deng and Laursen 1998; Gauthier et al. 2008), but to date, the exact physiological mechanisms have been unknown.

### AFGPs

AFGPs are a group of AFPs that developed in various polar and subpolar marine Teleost lineages, allowing their survival in frozen, cold seawater (Cheng 1998; Fletcher et al. 2001). AFGPs have long been found in several northern and Arctic Atlantic cod populations (Goddard 1994; Goddard et al. 1999; Hew et al. 1981).

The AFGPs were classified into eight subcategories, with the AFGP1 group containing proteins with the highest molecular mass (33.8 kDa) and the AFPG8 group containing proteins with the lowest (3 kDa) (DeVries et al. 1970). These proteins contain of 4 to 50 repeats of the Ala–Ala–Thr triad sequence, with a sugar branch (galactose N-acetylgalactosamine) at each hydroxyl group of the threonines' side chains. The recruitment and repetition of a short area spanning the boundary between the first intron and second exon of the trypsinogen gene results in the AFGP gene. This new section was then extended and iteratively replicated, yielding 41 tandemly repeated segments (Logsdon et al. 1997). The AFGPs' TH activity is related to the amount of repeating units: the larger AFGPs1–5 are significantly more active than the smaller AFGPs7 and 8 (Ahlgren et al. 1988; Burcham et al. 1984; Feeney and Yeh 1978; Kao et al. 1986; Knight et al. 1984). This variation is related to the difference in molecular weights, rather than just the substitution of proline in the repeating unit of the smaller AFGPs (Schrag et al. 1982). Even at low concentrations (~ 10^–12^ M), AFGP has been illustrated to highly inhibit ice recrystallization, suggesting that these proteins can be used in cryopreservation (Knight et al. 1984).

Employing NMR spectroscopy, the structural analysis of synthetic AFGPs with three repeating units revealed that these proteins folded into a left-handed helix comparable to the previously anticipated polyproline type II (PPII) helix (Bush and Feeney 1986; Bush et al. 1981). The disaccharide moieties are on the same side of the molecule in this synthetic AFGP structure, creating a hydrophilic face, while the hydrophobic face is packed into the other face of the molecule by the methyl groups of the Ala residues and the acetylmethyl groups of the GalNAc residues. This structure resembles that of the type I AFP's amphiphilic α-helix (Sicheri and Yang 1995), emphasizing the role of amphiphilicity in antifreeze function (Bush et al. 1981; Franks and Morris 1978). Natural resources are few and insufficient for the preparation of AFGPs for basic research or application research. Furthermore, substantial amounts of high-purity AFGPs are required for commercial use. Chemical synthesis has thus been studied for their manufacture. Chemical synthesis techniques for AFGP have proved difficult to establish, because it is a glycoprotein. Solution-phase synthesis (Anisuzzaman et al. 1988) and continuous flow solid-phase synthesis (Tseng et al. 2001) have also been explored. It has been proposed that the AFGPs precursor in notothenioids is a pancreatic trypsinogen, such as protease (TLP). Chen et al. (1997) and Cheng and Chen (1999) identified a chimeric TLP AFGP gene within the genome of the notothenioid *Dissostichus mawsoni* which produces AFGPs family. The gene was a hybrid exon that encoded seven AFGP molecules of different sizes and contained sections of the TLP gene. This exon 's position corresponded with a Ala–Ala–Thr present within the TLP genome, and the AFGP presumably formed as a consequence of unequal crossing over or replication slippage. Interestingly, the Arctic cod formed its AFGPs independently after divergence and this was possibly attributed to the decency of a different genomic predecessor (Chen et al. 1997).

#### Diatom AFPs

**Fig. 2:**
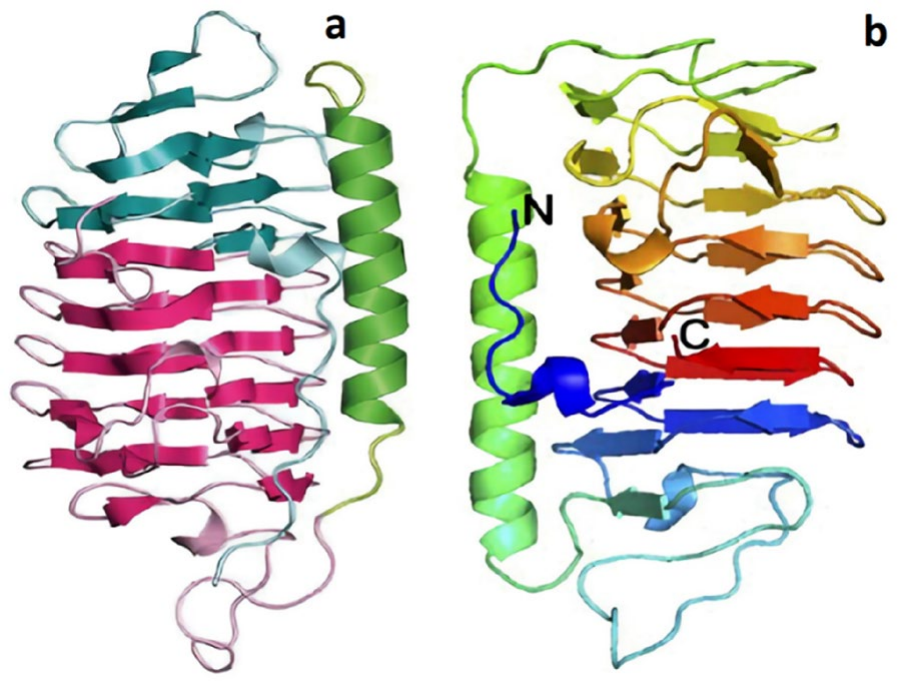
IBP Structure of **a** *Fragilariopsis cylindrus* IBP (fcIBP) (Kondo et al. 2018); **b** cn-AFP obtained using the modeller program (Gwak et al. 2014)

Polar sea diatoms have been identified as thriving under severe conditions in sea ice, with temperatures varying from − 1.8 to − 20 °C (Eicken 1992; Untersteiner 1986). Several studies have been performed to identify new AFP genes from polar sea diatoms (*Chaetoceros neogracile*, *Berkeleya sp*., *Navicula sp*., *Fragilariopsis sp*., and *Nitzschia frustulum*) and further research on gene expression has shown that AFP gene’s expression is regulated in reaction to stress factors, such as high salinity and cold temperature (Bayer-Giraldi et al. 2010, 2011; Janech et al. 2006; Krell et al. 2008; Uhlig et al. 2011). Consequently, AFP genes may play a significant role in the adaptation of diatoms to the environment. Gwak et al. (2010) first derived recombinant AFP (Cn-AFP) (Fig. 2b) from the marine diatom *Chaetoceros neogracile* and characterized its anti-freezing activity based on TH measurement and modified morphology of single ice crystals. Recombinant mature Cn-AFP displayed 16-fold higher TH activity than premature Cn-AFP at the same concentration. The ice crystal structure has been changed to an elongated hexagonal shape in the presence of the recombinant mature Cn-AFP, while single ice crystal had a circular disk shape in the absence of a Cn-AFP. Northern analysis was conducted to investigate the modulation of the steady-state Cn-AFP mRNA and revealed a dramatic accumulation of Cn-AFP transcripts when the cells were under freezing stress. This quick response to freezing stress and anti-freezing activity of recombinant Cn-AFPs indicates that Cn-AFP plays a significant role in the adaptation to low temperatures. Among the strategies proposed, a new class of ice-binding proteins (IBP) was characterized as the first molecular evidence for AFPs in diatoms (Janech et al. 2006; Krell et al. 2008). AFPs isolated from *F. cylinder* (fcIBP) (Fig. 2a) exhibited remarkable similarity to proteins present in the snow mold *Typhula ishikariensis* and the polar diatom *Navicula glaciei* (Janech et al. 2006; Hoshino et al. 2003).

A IBP with 25 kDa produced by the sea ice diatom *Navcula glaciei* (NagIBP) displayed effective recrystallization inhibition even at very low concentrations (from 10 μg/ml) (Janech et al. 2006).

#### Bacteria AFPs

Cold adapted bacteria undergo several biochemical adaptations to prevent freezing damage. The production of AFPs is one of these adaptation strategies. AFPs, when released, adsorb to the ice crystals and affect their growth trend, creating a shift in the ice microstructure, thus growing its porosity (Raymond et al. 2008). Increases the porosity facilitates the free passage of solutes and nutrients, thereby nourishing bacterial cells. Inhibiting the development of ice crystals also contributes to depression of the water freezing point, which prevents the cells from freezing injuries (Raymond et al. 2008). These AFPs are also expected to play a significant role in the survival of certain non-AFP producing species living in the cryoconite environment as well.

AFP isolated from Antarctic bacterium *Colwellia sp.* SLW05 (ColAFP) strain was homologous to AFPs from a broad range of psychrophiles. ColAFP attaches to many ice planes, involving the basal plane, according to fluorescence-based ice plane affinity research. These findings indicate that ColAFP is a hyperactive AFP. ColAFP's crystal structure at 1.6 Å resolution revealed an irregular β-helical structure, comparable to known homologs. Mutational and molecular docking investigations revealed that ColAFP attaches to ice via a compound ice binding site (IBS) centered on the β-helix's flat surface and the neighboring loop region. The IBS of ColAFP lacks the repeated sequences found in hyperactive AFPs. These findings show that ColAFP exhibits antifreeze activity via a compound IBS distinct from the IBSs shared by other hyperactive AFPs.

Pucciarelli et al. (2014) discovered two IBP sequences from a potential bacterial symbiont of the Antarctic psychrophilic ciliate *Euplotes focardii*, dubbed EFsymbAFP and EFsymbIBP. The AFP from the *Stigmatella aurantiaca* strain DW4/3–1, identified from the Victoria Valley lower glacier, is 57.43% similar to EFsymbAFP. EFsymbIBP is 53.38% similar to the IBP from the *Flavobacteriaceae* bacterium strain 3519–10, which was recovered from Lake Vostok glacial ice. At the amino acid level, EFsymbAFP and EFsymbIBP are 31.73% similar and are found on the same bacterial chromosome.

The low sequence identity and the tandem architecture, which appears to be unique to this symbiont, indicate to horizontal gene transfer (HGT). EFsymbAFP and EFsymbIBP are structurally similar to AFPs from the snow mold fungus *Typhula ishikariensis* and the Arctic yeast *Leucosporidium sp.* AY30. A phylogenetic study revealed that EFsymbAFP and EFsymbIBP cluster mostly with IBP sequences from other Antarctic bacteria, confirming the hypothesis that these genes correspond to an *E. focardii* Antarctic symbiont. These findings demonstrate that IBPs have a complicated evolving history that contains HGT events, most likely as a result of environmental stresses and the necessity for quick adaptation.

**Fig. 3:**
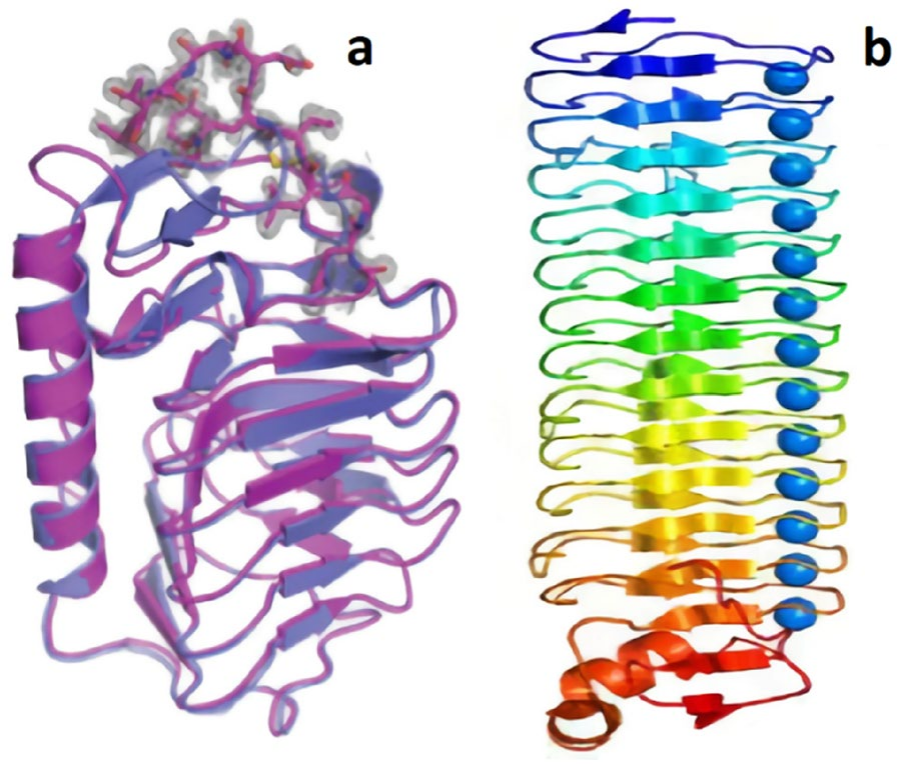
IBP X-ray crystal structures. **a** *Flavobacterium frigoris* PS1 (FfIBP) (Do et al. 2014); **b** *Marinomonas primoryensis* (MpAFP) (Garnham et al. 2008)

According to Mangiagalli et al. (2017), the recombinant EfcIBP can withstand freezing without causing functional damage and is moderately heat stable, with a midway temperature of 66.4 °C. EfcIBP has an interesting mix of features not seen in other bacterial IBPs when tested for ice effects. When kept at a constant temperature inside the TH gap, EfcIBP shows TH activity (0.53 °C at 50 µM) and can halt a crystal from developing. When exposed to cold temperatures, EfcIBP protects purified proteins and bacterial cells from freezing damage. EfcIBP also has a DUF3494 domain, which is shared by secreted IBPs, and a putative N-terminal signal sequence for protein transport. These characteristics suggest that the protein is either attached at the cell's outer surface or clustered surrounding cells to offer a survival benefit to the whole cell consortium. The crystal structures of IBPs from *Flavobacterium frigoris* PS1 (FfIBP) and *Marinomonas primoryensis* (MpAFP) are shown in Fig. 3.

#### Fungal AFPs

**Fig. 4:**
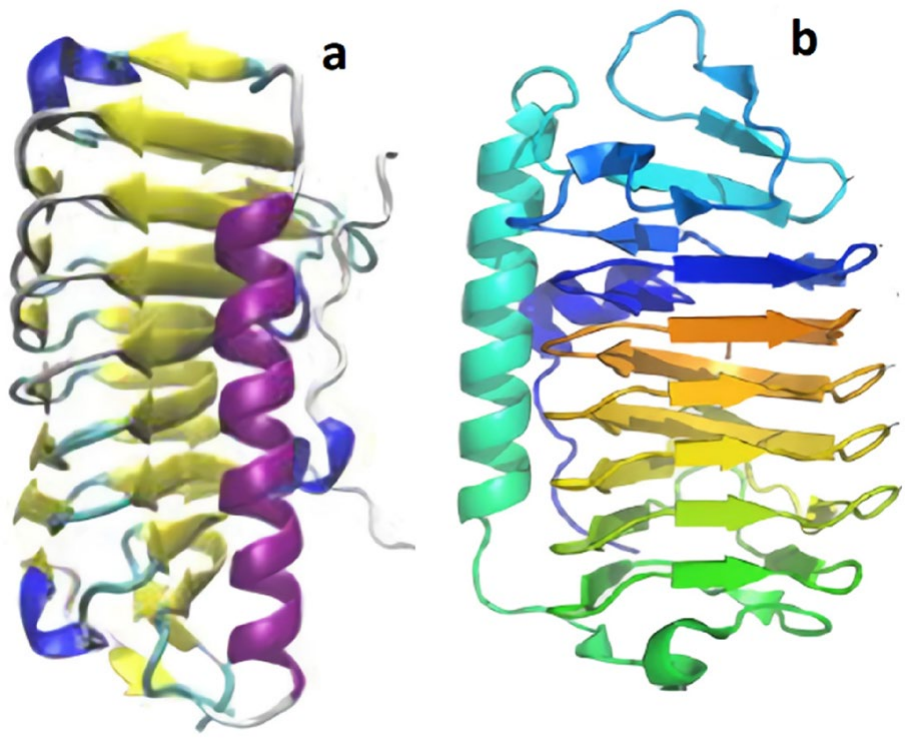
Structures of IBP. **a** LeIBP isolated from psychrophilic yeast *Glaciozyma sp*. AY30 (Kim et al. 2014); **b** *Typhula ishikariensis* (TisIBP6) (Kondo et al. 2012)

Present knowledge of cold-adapted fungi has demonstrated that, even in the most extreme cold ecosystems, there are several metabolically active fungal species. To survive under these intense conditions, fungal strains have developed a variety of living strategies and functions intended to perform different ecological roles. Many psychrophilic and *Basidiomycetous* psychrophilic yeast species have been screened and confirmed to have anti-freezing activities. Just two mushrooms (*enoki* and *shiitake*), one snow mold fungus (*Typhula ishikariensis*), and two yeast species (*Glaciozyma antarctica* and *Glaciozyma sp.* AY30) were characterized for their genetic and antifreeze properties (Fig. 4) (Lee et al. 2010; Raymond et al. 2009; Singh et al. 2014; Xiao et al. 2010).

Lee et al. (2010) discovered the first antifreeze action of a non-canonical AFP in the Arctic yeast *Glaciozyma sp*. AY30 (designated as LeIBP, accession no. ACU30806). *Glaciozyma sp.* AY30 is a cold-adapted psychrophilic yeast isolated from the frozen Tvillingvatnet, a freshwater pond near the Arctic Dasan site in Norway's Svalbard island. LeIBP is comprised of 261 amino acids and has one N-glycosylation site and an N-terminal signal sequence. The presence of a signal sequence was confirmed by the presence of a LeIBP band (approximately 25 kDa) and TH activity in the culture medium (Park et al. 2012).

The LeIBPs contain a dimeric right-handed β-helix fold composed of three components: a large coiled structural domain, a prolonged helix region (residues 96–115 form a long α-helix that packs along one face of the β-helix), and a C-terminal hydrophobic loop region (^243^PFVPAPEVV^251^). The C-terminal hydrophobic loop region forms entangled dimer connections and has an expanded conformation pointing away from the coiled structural domain's body. Furthermore, structural study of glycosylated LeIBP with sugar moieties linked to Asn^185^ offers a foundation for glycosylated LeIBP's improved stability and secretion. Using site directed mutagenesis, it was also revealed that the aligned Thr/Ser/Ala residues are important for ice-binding inside the B face of LeIBP (Lee et al. 2012).

Hashim et al. (2013) used the cloning of a cDNA encoding the AFP1 to demonstrate the strong antifreeze activity of AFP generated by the psychrophilic yeast *Glaciozyma Antarctica* PI12. During a genome sequence survey of the genome of *G. antarctica*, a novel AFP gene was found. The cDNA encoded a 177-amino acid protein that shared 30% of its amino acid sequence with a *Typhula ishikariensis* fungus AFP. AFP1 expression levels were measured using real-time quantitative polymerase chain reaction (RT-qPCR), and the maximum levels were seen within 6 h of growth at -12 °C. The AFP's cDNA was cloned into an *Escherichia coli* expression system. Recombinant Afp1 expression in *E. coli* resulted in the production of inclusion bodies, which were then denatured with urea and allowed to refold in vitro. The AFP capabilities of recombinant Afp1 were validated by activity testing, which revealed a high TH value of 0.08 °C. The amino acid sequence analysis revealed that AFP1 has four α-helices. Each helical peptide demonstrated antifreeze activity, according to Shah et al. (2012).

## Diversity of marine AFPs structure and evolution

AFPs provide a large structural variability for a group of proteins that have the same role. The crystal structures of the AFPs consist of a single α-helix, a four-helix group, multiple single β-solenoids with different cross sections, a polyproline type II helical group, and two mixed-structured globular folds.

At temperatures below zero, AFPs are generated, folded, and functioned. AFPs have remarkably diverse folds that are more dependent on hydrogen and disulfide bonds. The bacterial AFP from *M. primoryensis* derived from a brackish, ice-covered lake in Antarctica is stabilized by an inner row of 13 Ca^2+^ ions on one side of the core (Garnham et al. 2011). The charge on these divalent metal ions is partially neutralized by the Asp side chains pointing inward.

This variety of AFP sequences and structures indicates that they have developed separately in many biological kingdoms. Evolutionary survival pressure in ice-laden niches has led to virtually equal development of AFGPs in two groups of fish (cods and notothenids) from the northern and southern hemispheres, respectively (Chen et al. 1997). In fact, in northern hemisphere fish, the alanine-rich single α-helix (type I AFP) has evolved four times independently (Graham et al. 2013). The lateral transfer of the AFP genes was another outcome of the intense selective pressure to control ice growth. For example, the gene for one AFP, which is the triangular β-solenoid structure supported by the flanking α-helix, has been distributed among microorganisms, such as bacteria, fungi, yeasts, diatoms, algae, and even a crustacean (Bayer-Giraldi et al. 2011; Kiko 2010). An even more amazing lateral gene transfer has contributed to the presence, in both herring and smelt (fishes of two separate superorders) of a lectin-like type II AFP (Graham et al. 2008; Guo et al. 2012). The existence of remarkably different AFPs in closely related organisms indicates their recent geological time acquisition. The presence of AFPs in Teleost fishes is well associated with the ones of glaciation at sea level in the Cenozoic era 20–40 million years ago (Fletcher et al. 2001). Therefore, the current distribution of AFPs in organisms appears to have been the result of a combination of independent evolutionary events, either with convergence to a common structure, or evolving through lateral gene transfer.

Despite their differences in overall structure, IBPs share a flat, relatively hydrophobic face that serves as the protein's IBS. The IBS has been predicted (Nutt and Smith 2008; Gallagher and Sharp 2003) and demonstrated (Garnham et al. 2011) to guide water molecules into an ice-like lattice, which integrates with the quasi-liquid layer on ice freezing the IBP onto its surface. IBP freezing adsorption to ice creates a microcurvature of the crystal surface, making it thermodynamically harder for water molecules to build up and establish the crystal (Knight 2000; Kuiper et al. 2015). It has been suggested that AFP binding to ice surfaces is likely to be irreversible (Kuiper et al. 2015; Raymond and DeVries 1977; Celik et al. 2013). This is because ice-binding by IBPs is a diffusion-controlled reaction, with the rate-limiting phase being the IBP coming into contact with the ice and making an effective bonding connection. For the latter to occur, enough ice-like waters on the IBP's IBS must merge with and freeze to the quasi-liquid layer surrounding ice. Two strategies have been used to increase the number of ice-like waters. The one is to increase the surface area of the IBS; the other is to connect a number of IBSs that can bind ice at the same time. The former strategy manifests in nature in natural isoforms with extra sequence repeats that increase the surface area of the IBS (Schrag et al. 1982; Wu et al. 2001; Leinala et al. 2002; Mok et al. 2010). By increasing the number of helical coils, this strategy has been mimicked for a β-solenoid AFP (Marshall et al. 2004).

The second strategy, having multiple IBS, occurs in nature as well, with a natural dimer of type III AFP from the Antarctic eel pout (*Rhigophila dearborni*) (Wang et al. 1995b). Nishimiya et al. (2003) investigated the activity of type III AFP linear multimers by recombinantly linking monomers and dimers to form a chain up to four AFPs long. These researchers discovered an increase in TH activity on both a molar and domain basis. In another application of this approach, a recombinant form of the single α-helix type I AFP was conjugated to the primary amines of polyallylamine chains via its C-terminal end (Can et al. 2011). The effects of AFP multimeric conjugation have also been explored by conjugating the globular 7 kDa HPLC12 isoform of type III AFP from the ocean pout (*Macrozoarces americanus*) through a C-terminal cysteine residue to a second-generation polyamidoamine (PAMAM) dendrimer utilizing a heterobifunctional cross-linker. When compared to monomeric AFP (Stevens et al. 2015), AFPs conjugated to polyallylamine chains and PAMAM dendrimers increased TH activity by twofold and more than fourfold, respectively. AFPs bound to PAMAM dendrimers inhibited ice-recrystallization 8–10 times more.

## Physicochemical properties of marine AFPs

### Thermal Hysteresis

The mechanism of TH activity is considered to be an adsorption–inhibition mechanism that binds to ice surfaces and inhibits ice crystal growth (Raymond and DeVries 1977; Raymond et al. 1989; Knight et al. 1991; Dalal and Sonnichsen 2002; DeVries 1986). When AFPs bind to ice, they lower the local freezing point by pressuring ice to grow in curved fronts between the bound AFP molecules (DeVries 1986; Wilson et al. 2006).

TH has been used to quantitatively clarify the AFPs' activity. For most fish AFPs, the noticed TH activity is about 1 °C (Fletcher et al. 2001; Davies 1990). This temperature gap enables polar fish to survive in cold environments by ample insulation facing seawater during the winter season (− 1.9 °C). Many marine AFPs are associated to sea ice as well as to fish AFPs (Bayer-Giraldi et al. 2011; Do et al. 2014; Hanada et al. 2014; Janech et al. 2006; Jung et al. 2014, 2016; Kiko 2010). In contrary to blood plasma in polar fish seawater in brine channels in sea ice undertake freezing to ice. Consequently, AFPs obtained from sea ice-associated bacteria, microalgae, and eukaryotic protists are secreted into the surrounding environment to keep themselves from freezing and so, to avoid damages they must show a high TH. FfIBP is an IBP encoded by the Antarctic bacterium *Flavobacterium frigoris* PS1. Sequence alignments and structural comparisons of IBPs caused two groups of IBPs to be specified, depending on sequence differences between the α2 and α4 loop regions and the presence of the disulfide bond. Although FfIBP closely resembles *Leucosporidium* (recently re-classified as *Glaciozyma*) IBP (LeIBP) in its amino acid sequence, the TH activity of FfIBP appears to be tenfold higher than of LeIBP TH (Do et al. 2014).

While ice adhesion proteins have similar activities to those of AFPs, they serve as a means to bind to the ice and not specifically to block its growth (Guo et al. 2012). Adsorption–inhibition model is an accepted model to clarify the inhibition of ice growth by AFPs. This model describes ice growth as happening in the intervals among adsorbed AFPs (Raymond and DeVries 1977). The process improves ice surface curvature between bound AFP molecules, consequently diminishing its radius from an infinite to a finite severity. According to the Gibbs–Thomson effect, suggesting that a solid's melting point of equilibrium is related to the curvature of the solid surface and the interfacial energy, such growth in the surface curvature leads to depression of the freezing point energy (Knight et al. 1984; Yeh and Feeney 1996). Although the adsorption–inhibition model assumes irreversible binding of AFPs to ice, the irreversibility of this binding remains the subject of the recent debate in the AFP community (Celik et al. 2013; Meister et al. 2013). Furthermore, while the dependence of TH activity on the AFP concentration has been extensively studied, the time dependence of this process remains unknown. Chapsky and Rubinsky (1997) illustrated that the TH activity of a solution of AFP I from winter flounder, *Pseudopleuronectes americanus*, could increase over time. The same researchers found that after exposure of an ice crystal to the solution of AFP I, TH increased by a factor of 5 overtime and reached a plateau 60–180 min, depending on the concentration. They theorized a time-dependent TH due to the accumulation of AFP molecules on the ice surfaces or the readjustment of adsorbed AFP molecules on the ice (or a combination of the two). Kubota (2011) suggested an AFP ice growth inhibition model for the kinetics. This model assumed gradual and temporary adsorption of AFPs to ice; therefore, it took a period to achieve an adsorption equilibrium. This model's working assumption was that the AFPs adsorbed reversibly to ice. Applying ellipsometry techniques based on optical shifts in the water–ice interface, Wilson et al. (1993) measured the accumulation of AFGPs on ice crystal surfaces. They found the interface signal was changing over time until it reached a plateau at about 60 min. Their analysis was that the alteration at the interface of water ice was affected by the adsorption of AFGP molecules to ice crystal planes.

The TH activities of the IBPs are diverse (Table 1), ranging from, 0.08 °C (at 200 µM of Afp4 from *Glaciozyma antarctica*) (Hashim et al. 2014) to 3.8 °C (at 140 µM of *Colwellia sp*. strain SLW05 IBP (ColIBP)) (Hanada et al. 2014). Although these measurements were made with various buffers with different ionic strengths and are not directly comparable, variations in laboratory techniques are unlikely to explain such a large disparity in activity. IBP isoforms from the same species, such as isoforms 6 and 8 of TisIBP from the snow fungus *Typhula ishikariensis*, can exhibit a wide range of TH activity. Despite its great sequence similarity (83.4%), TisIBP6 and TisIBP8 have TH activity of 0.3 and 2.0 °C at 0.11 mM, respectively.

**Table 1:** TH activity of ice-binding domains

Organism	Specific name	TH (°C) (AFP concentration)	MW (kDa)	References
*Shewanella frigidimarina*	SfIBP_1	2 (80 µM)	24.5	Vance et al. 2018
*Flavobacteriaceae* bacterium 3519–10	IBPv	2 (50 µM)	54.0	Wang et al. 2016
*Brachyopsis rostratus*	Fish type II (Ca^2+^-independent)	0.5 (0.25 mM)	14	Nishimiya et al. 2008
*Clupea harengus*	Fish type II (Ca^2+^-dependent)	0.4 (0.55 mM)	14	Liu et al. 2007
Bacterium consortium of *Euplotes focardii*	EfcIBP	0.53 (50 µM)	23.4	Mangiagalli et al. 2017)
Fish antifreeze glycoproteins1–5	Fish AFGP1–5	0.2 (0.04 mM)	15	Olijve et al. 2016
*Typhula ishikariensis*	TisPB6	0.32 (140 μM)	22.21	Cheng et al. 2016
*Typhula ishikariensis* 23	TisPB8	2 (180 μM)	22.3	Kondo et al. 2012
*Leucosporidium *sp. AY30	LeIBP	0.35 (370 μM)	26.2	Lee et al. 2012
*Chaetoceros neogracile*	CnIBP	0.80 (40 µM)	26.2	Gwak et al. 2010
*ColAFP Colwellia *sp. SLW05	ColAFP	3.8 (0.14 mM)	26	Hanada et al. 2014
*Flavobacterium frigoris* PS1	FfIBP	2.5 (50 µM)	28.4	Do et al. 2014
*Glaciozyma antarctica*	Afp4	0.08 (200 µM)	25.3	Hashim et al. 2014
*Antarctomyces psychrotrophicus*	AnpIBP1	0.56 (150 µM)	21.4	Arai et al. 2018
*NagIBP Navicula glaciei*	NagIBP	3.20 (1.6 mM)	24.4	Xiao et al. 2014
*Fragilariopsis cylindrus*	fcIBP11	0.90 (350 µM)	25.9	Bayer-Giraldi et al. 2011

### Inhibition of Ice Recrystallization

IRI is another feature of AFPs defined as the noticeable decline of the ice texture change during the annealing process just under the sample melting temperature (Budke et al. 2014; Knight et al. 1986). Ice recrystallization involves ice grain boundary migration (Knight et al. 1986), in which large ice crystals increase in size, and small crystals vanish, and AFPs prevent grain boundary migration mechanisms by preventing ice from growing and melting at the boundaries (Knight et al. 1988, 1995). The mechanism of ice recrystallization is related to Ostwald ripening, in which the diffusion of free H_2_O molecules among ice crystals plays an important role. AFP adsorbed on the ice surface can hinder ice recrystallization by restraining H_2_O diffusion (Ishibe et al. 2019). IRI improves organisms' freezing resistance in environments, where only small ice crystals occur. According to some reports, numerous macromolecules exhibit IRI activity, but only AFPs have the TH property (Ding et al. 2015). Due to the IRI activity, when AFP is added to a sample, the produced ice crystals are very small. These small ice crystals, on the other hand, can combine to create large aggregations and fuse if the curved sections of two ice crystals get too near together. Since the system becomes less stable as curved ice grows, the ice crystals may eventually form a network structure to stabilize the system (Kaleda et al. 2018).

IRI is already important at concentrations of sub-micromolar AFP that are below concentrations needed for TH (Tomczak et al. 2003). Ice crystals remain small as a result of IRI, which is necessary for survival in cold conditions (Venketesh and Dayananda 2008). Knight et al. (1995) have extensively reported the TH inhibitory effect of AFPs on the recrystallization of ice. AFP from winter flounder can inhibit crystallization in ice when enough amount of liquid is present and the system contains salts and the temperature is not so low. In this case, the AFP binds to the ice surface at the ice solution interfaces in grain boundaries, avoiding migration of the solution and effectively immobilizing the boundaries, since the concentration of salt required to induce recrystallization inhibition effects; therefore, AFPs could play a role in the survival of organisms by preventing damages due to recrystallization.

Winter flounder AFP has the power to block ice crystallization when there is enough liquid, and the device contains salts, so the temperature is not so low. In this case, the AFP links to the ice surface at the ice fluid interfaces in grain boundaries, avoiding migration of the solution and effectively inactivating the boundaries, because the salt concentration needed to cause recrystallization inhibition effects; therefore, AFPs might play a role in organism survival by preventing recrystallization loss (Knight et al. 1995).

AFPs are modifiers of the growth of macromolecular ice crystals that safeguard body tissues against freeze injury (DeVries and Wohlschlag 1969; Fletcher et al. 2001; Gwak et al. 2014). AFPs adsorb onto the surface of ice crystals and thus act as a defilement on the surface of ice crystals. As a consequence, the AFPs meddle with the connection and disconnection of water molecules on ice grains, which fully detains the grain boundary migration.

One major barrier to the improvement of potent IRI active compounds is the absence of a reliable, high-throughput IRI quantification method. Thin ice films with plainly visible grain boundaries are the prime prerequisite for a robust IRI operation assessment. This need contributed to the creation of the splat cooling system (Knight et al. 1988).

### Dynamic Ice Shaping

By definition, the ability to adsorb to one or more ice planes is the basic property of all IBPs. The shape or faceting of ice crystals is one of the earliest signs of the presence of an IBP, even at micromolar quantities concentrations (Knight et al. 1984), where TH is still too low to quantify. IBPs tend to bind to at least one of the crystal planes found in ice. Therefore, IBPs use an adsorption–inhibition mechanism (Knight, 2000) to slow down ice development in the area, allowing more of the target plane to be used for additional IBP binding, resulting in the formation of a facet.

In most cases, ice crystals grow uniformly in all planes, except the basal plane, resulting in the production of a disc-like shape. AFPs, on the other hand, have the potential to adsorb onto certain ice planes. The AFP bound planes exhibit micro curvatures between ice bound-AFP molecules, making subsequent ice crystal development thermodynamically less favorable (Davies and Hew 1990; Hew and Yang 1992). A distinct ice crystal shape emerges from such ended growths towards particular ice planes. Type I and type II AFPs cause hexagonal bipyramid shaped ice crystals to form (Davies et al. 2002; Takamichi et al. 2007). Maxi, on the other hand, causes the creation of lemon-shaped ice crystals (Graham and Davies 2005).

The capacity of an antifreeze active compound to affect the morphology of a particular ice crystal is known as DIS. If a TH gap exists, the morphology is usually specified within this band or as close to the freezing point as possible. Temperatures below this point can cause explosive development of crystals on particular faces. DIS is a macroscopic phenomenon that occurs when the crystal habit of ice crystals varies on a molecular level. These will be discussed in tandem, with the appropriate name being used depending on the size at which the process is being investigated. While there is no uniform definition, it is essential to distinguish between data analysis and interpretation approaches. This can be visualized using a nanolitre osmometer (Chakrabartty et al. 1989) to observe the growth of a single ice crystal, hemispherical etching, (Knight et al. 1993) formation of pits during ice growth (Raymond et al. 1989), or wide-angle X-ray diffraction (Mastai et al. 2001).

The mechanism of DIS is supposed to be that AF(G)Ps attach to specific planes of ice (typically the major prismatic plane), restricting the attachment of additional water molecules to the ice crystal face and so impeding crystal growth along the bound axis (Gibson et al. 2010). The development of long, spicular needles along the unbound axis leads in destruction to biological matter on a cellular level, bursting and exploding cells by effective puncturing (Carpenter and Hansen 1992). However, because DIS happens only below the TH gap during ice crystal formation, this trait is not harmful to marine species that relies on AFGPs for survival (Gibson et al. 2010; Biggs et al. 2017).

### Interaction with membranes

At low temperatures, AFPs can also act by interacting with the plasma membrane, as shown for AFP I in liposomes, by prevention of ion leakage across the membranes or blocking ion channels (Tomczak et al. 2002; Venketesh and Dayananda 2008), allowing these species to survive in waters that are colder than the freezing point of their body fluid (Inglis et al. 2006). AFPs appear to bind to the phospholipid bilayer, raising and stabilizing the membranes' phase transition temperatures. Rubinsky et al. (1990) illustrated AFPs' power to defend cell membranes from hypothermic harm. In addition, AFPs have been proved to protect liposome leakage, since they were cooled through their change phase temperatures (Hays et al. 1996; Wu et al. 2001).

These findings demonstrated that the AFPs interact with the cell membrane's lipid bilayer. One reason for cold-induced cellular harm may emerge when cells are cooled by the lipid membrane thermotropic phase transition temperature. Throughout this phase transition from liquid crystalline to gel, membranes become permeable, resulting in the loss of intracellular material and penetration of extracellular materials. Research on human blood platelets showed that AFPs could prevent cold-induced morphological changes initiated when the platelets passed through their phase transition temperature, which supports this hypothesis. Model membranes with different concentrations, such as dielaidoylphosphatidylcholine (DEPC), dielaidoylphosphatidylethanolamine (DEPE), and dielaidoylphosphatidylglycerol (DEPG), have been used to examine the nature of the interactions between AFPs and cell membranes, since the transition temperature of each lipid depends on the degree of unsaturation of lipid tails and the number of carbons in the lipid alkane chains (Kun et al. 2009; Hays et al. 1996, 2001, 2002, 2003; Kun and Mastai 2010; Tablin et al. 1996; Tomczak et al. 2001; Wu et al. 2001).

## Purification of marine AFPs

The use of protein/peptide from natural sources requires a purification process from complex mixtures (with a previous extraction process if the protein is in a solid matrix). As cells must be disrupted to isolate intracellular proteins in soluble form from its intracellular compartment, appropriate techniques for cell disruption must be applied. This disruption technique should be as gentle as possible to the protein as the starting point for all subsequent processes is the extraction step.

Purification can be achieved using typical methods, such as column chromatography, ammonium sulfate salt precipitations, antibody affinity column chromatography. The use of ion-exchange chromatography as described by DeVries and Lin (1977) is a recommended method of purification of naturally occurring polypeptides (e.g., formed in fish) and purifying AFP from winter flounder, *Pseudopleu-ronectes americanus.* GmAFP IV was purified from Pacific cod (*Gadus macrocephalus*) by Ni–NTA affinity chromatography and observed by electrophoresis of SDS-PAGE (Mao et al. 2018).

**Fig. 5:**
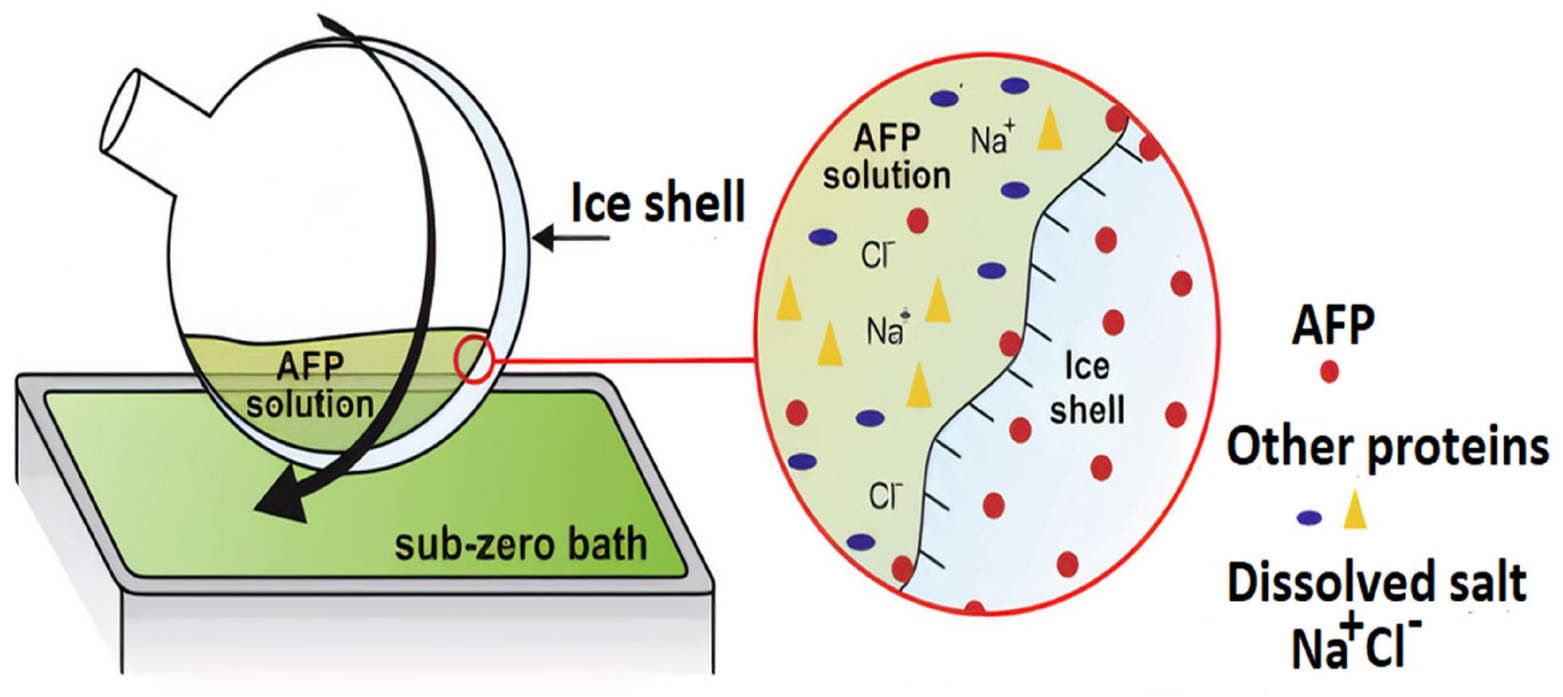
Ice-affinity purification of IBPs using an ice-shell (Marshall et al. 2016). Apparatus used to capture IBPs from diverse mixtures of proteins and other solutes. In this system, the separating surface area was improved by isolating the IBPs into an ice shell formed within a rotating round-bottom flask, partially immersed in the sub-zero bath. In general, each ice-binding compound can be recovered from the liquid solution

The useful tool for the purification of AFPs is ice affinity purification (Kuiper et al. 2003; Raymond and Knight 2003) (Fig. 5). In one type of this procedure, a small ice mass is frozen in a cold-finger brass (ethylene glycol circulates form a refrigerated bath), and then it is placed in a beaker containing an extract with the unknown AFP. As the center is cooled the ice expands gradually. Most proteins are removed from the ice front, but AFPs adsorb to the surface of the ice and become overgrown with new ice layers. AFPs are recovered from the cold-finger by melting the ice mass and trace impurities can be excluded from the melt by growing a new ice mass. After, applying only three cycles of ice affinity purification, pure AFP can be derived from a crude homogenate of the starting organism (Graham and Davies 2005). This technique’s main advantage is that it is not necessary to know anything about the physical properties of the AFP (including molecular weight, isoelectric point, hydrophobicity or posttranslational modification) to complete the purification process.

Falling water ice affinity purification (FWIP) is a new ice affinity purification method for efficient and high yield purification of large volumes of AFPs. As in other methods of purification of ice affinity, FWIP did not need protein affinity tags and applies to various types of IBP solutions including hemolymph fluid. In this method, a crude IBP solution is allowed to flow through a commercial ice machine's chilled vertical surface. The surface temperature is slowly lowered until ice crystals are formed, to which the IBPs bind, but other solutes do not bind. It was observed that a maximum of 35 mg of IBP incorporated into 1 kg of ice. Two rounds of FWIP resulted in a purity of > 95%. An ice machine generating 60 kg of ice per day can be used for the purification of one gram of IBP per day. In conjunction with the efficient concentration of protein solution through tangential flow filtration, the FWIP can be used to attain grams of IBP. The production of a larger amount of AFP may ease future research as well as their potential application in cryopreservation, medicine, and other areas (Adar et al. 2018).

## Mechanisms of action of marine AFPs

Crystallization involves two major steps: nucleation and propagation of ice crystals by the growth of the nucleus. Nucleation normally happens around a foreign molecule (heterogeneous nucleation) or through a process (homogeneous nucleation) in the case of absolute pure water. Ice recrystallization happens when the temperature fluctuates within the subzero range by various mechanisms (Hassas-Roudsari and Goff 2012).

The properties of AFPs include TH (Meister et al. 2013; Takamichi et al. 2007), modifying ice crystal morphology (Chapsky and Rubinsky 1997; Meister et al. 2013), inhibiting ice crystal growth (recrystallization) (Koh et al. 2014; Shah et al. 2012; Kim et al. 2014), enhancing cellular integrity and reducing microbial growth. While the first three properties are related to interactions between AFP, water, and ice, the other properties occur at temperatures slightly above the system freezing point (Boonsupthip and Lee 2003).

AFPs have several mechanisms to protect against ice. These include the reduction of the point, where ice crystals grow (reduce the freezing point but not the melting point, the so-called TH effect), but also modification of the crystallization of ice so that smaller crystals and crystals of different shapes are produced (Crevel et al. 2002). AFPs use a non-colligative mechanism to exert their freezing point depression activity (Crevel et al. 2002; Davies et al. 2002), which minimizes their impact on the osmotic pressure of the fish plasma (Crevel et al. 2002).

AFPs seem to exert their effect by accumulating at the water–ice interface and thus changing the growth of crystals (Crevel et al. 2002; Kubota 2011). On a macroscopic level, the mechanism is considered to be an adsorption–inhibition process in which the biological antifreeze binds to the growing ice crystal surface (Davies et al. 2002; Hassas-Roudsari and Goff 2012; Kubota 2011; Nutt and Smith 2008; Raymond and DeVries 1977; Smolin and Daggett 2008). According to this model, crystal growth occurs between adjacent antifreeze molecules on ice surfaces, and these surfaces grow with high surface curvature (Bouvet and Ben 2003; Davies et al. 2002; Hassas-Roudsari and Goff 2012). As the energetic cost of applying a water molecule to this convex surface is high, the freezing point depression is not an equilibrium, while the melting point stays stable. It is referred to as the Kelvin effect and the TH (Hassas-Roudsari and Goff 2012; Nutt and Smith 2008). Two models rationalize the inhibition of ice growth based on two or three-dimensional Kelvin effect; the model of mattresses and the model of step pinning. The adsorbed molecules in the mattress model prevent ice growth perpendicular to the ice surface, while the molecules in the step-pinning model block ice growth (Bouvet and Ben 2003).

## Applications of marine AFPs

AFPs are suitable for the food industry, agriculture, biotechnology, medicine, and cryopreservation of cells, tissues, and organs (Knight and Duman 1986; Koushafar and Rubinsky 1997; Modig et al. 2010). Research experiments have shown that AFPs act as cryoprotectants in cryogenic and hypothermic preservation of isolated organs, tissues, cells, sperm, oocytes, embryos, and RBCs (Budke et al. 2009; Chao et al. 1996; Venketesh and Dayananda 2008). AFPs can also be used for the development of ice models in material sciences and in coatings to avoid ice forming (Abraham et al. 2015; Gibson 2010). The gap between the apparent potential of AFPs and potential industrial applications is due to the lack of processes to purify AFPs efficiently and affordably. The availability of large quantities of purified AFPs is crucial for the industry, especially in cryopreservation, where low protein content hinders the development of practical technologies and restricts basic research. Food applications are mostly found either in patents or in a few publications. Unilever has set up a new research and development system complemented by external partners to investigate AFPs features and its application in ice cream to inhibit the production of ice crystals. This technique was applied to the ice cream, where ice crystal development has been very limited relative to the control (Warren et al. 1992). Commercial ice cream products with AFPs are currently on the market (Crilly et al. 2008). In ice cream, a small quantity of AFPs was applied to the thawed samples and then the samples were frozen at about − 80 °C and kept at a temperature between − 6 °C and − 8 °C for various timeframes. Microscopic changes in recrystallization have been observed. Another ice cream product was the Eskimo brand Twin Pop (Banana flavored) distributed (Los Angeles, CA) by Tomorrow products. After 1 h at − 6 °C to − 8 °C, the control sample had significantly larger crystals, while the AFP sample did not display any crystal growth. The other ice cream evaluated was a Merrit Foods products (Kansas City, MO). This ice cream was a combination of a root beer shell and a core of vanilla ice cream. The AFP sample showed very little ice-crystal growth after 1 h, while the control sample displayed a definite increase in the ice-crystal size (Warren et al. 1992). It has been proposed that the use of AFPs in meat may minimize drip loss (Inglis et al. 2006; Rubinsky et al. 1990). Besides, due to their ability to inhibit ice recrystallization, AFPs have been used to reduce damage to RBC undergoing freeze–thaw cycles (Kang and Raymond 2004) or destroy harmful cells during cryosurgery by freezing, regardless of the thermal parameters used (Koushafar and Rubinsky 1997).

### Food applications

#### Safety Aspects for the introduction of marine AFPs into food products

AFPs can be used in foods that are frozen only for preservation. The incorporation of novel proteins into food products may cause the risk of an allergic reaction in individuals who are sensitive to this protein (Bindslev-Jensen et al. 2003).

The role of AFPs in fish as potential food allergens has not been reported. The consumption of AFPs in the diet is likely to be significant in most northerly and temperate regions. Most of this consumption is expected to be from edible plants, considering their importance in the diet, but in some regions, the consumption of fish may be also important. As far as it can be known, AFPs are consumed with no signs of negative health effects, in both short or long term, at levels not exceeding 0.01% by weight and more commonly less than 0.005% that are proposed to be used in products (EFSA 2008). The history of AFPs consumption is that their functional properties do not impart any toxicologically significant effects, such as inhibition of cholinesterase (Crevel et al. 2002). Although, historically, AFPs intake is regarded as healthy for the incorporation of proteins into food products an investigation of potential allergic reactions, is a prerequisite for the marketing of food. However, it is not possible to determine whether a protein is an allergen solely based on its physical or chemical properties. Baderschneider et al. (2002) compared the sequence of AFP type III (HPLC12 fraction from ocean pout) with known allergens and pepsin resistance and they observed that the type III protein, or any heptamer peptides derived from it, showed no sequence similarity with known allergens. Moreover, pepsin quickly hydrolyzed the type III and type III glycoconjugates, resulting in fragments with a molecular weight of less than 4 kDa. Therefore, they reported that type III, its glycoconjugates, and pepsin hydrolysis products were unlikely to provoke allergic reactions in individuals sensitized to known allergens or provoke an allergic reaction in unsensitised individuals.

#### Food preservation

For some foods, the freezing process may lead to a decrease in either food quality or nutritional value. Foods that are consumed after thawing, including, but not limited to, meats are prone to suffer damage of cell membranes due to intracellular ice formation. This results in water loss and causes a lower quality of food products following thawing (a loss of nutrients and a reduced capacity of water holding). Foods that are consumed in a frozen state, such as ice cream, may also have their quality degraded over time by the development of texture compromising ice crystals.

Adding low concentrations of AFPs to food products can promote inhibition of ice recrystallization, which can improve food quality and nutritional value as well. A reduction in the size of the ice crystal found in meats has been reported following treatment with either fish AFPs or AFGPs, since small ice crystals will typically cause less damage to the cells than large ones, adding a higher concentration of AFPs can also be beneficial to foods that shouldn't be frozen at all. The higher concentration of AFPs will enhance inhibition of ice crystal growth, thereby enabling foods such as strawberries to be stored at low temperatures with a reduced risk of loss of quality.

AFPs are not a major fish allergen and the bioinformatic sequence homology analysis did not show similarity to known allergens (EFSA 2008). The main issue that must be discussed in developing a cold storage system for food products containing AFPs is how AFPs may be incorporated into food. Since AFPs work extracellularly, it is also possible to incorporate AFPs directly into food through mechanical methods, mixing, injecting, soaking, or vacuum-infiltration. This allows the use of AFPs in a wide variety of foods. The use of AFPs to inhibit ice growth and some evidence exists that the use of specific AFPs under some conditions can actively damage cellular structures (Hincha et al. 1993). Several possible food applications of fish AFPs are presented in Table 2.

**Table 2:** Potential food applications of fish AFPs

AFP source	Type of AFPs	Added to product	AFP Quantities	Function	References
Antarctic cod (*Dissostich-usMawsoni*)	AFGP (1–8)	Soaking beef	0–1 mg/ml	Reducing the ice crystals size	(Payne et al. 1994)
Winter flounder (*Pseudo-pleuronectes americanus*)	I				
Grubby sculpin (*Myoxocephalus aenaeus*)	I	Frozen dough	─	Improving fermentative efficiency	(Panadero et al. 2005)
Winter flounder (*Pleuronectes americanus*)	I	Ice cream	1 µg/ml	Inhibition of ice recrystallization	(Gaukel et al. 2014)
Ocean pout (*Macrozoarces americanus*)	III				
Rock cod (*Gadus ogac*)	AFGP				
Eelpout (*Zoarces elongates Kner*)	III	Bovine embryos	10 mg/ml	High viability	(Ideta et al. 2014)
Antarctic Cod	AFGP	Lamb meat	0 µg, 0.01 µg, 1 µg, 100 µg/kg live weight	Reducing drip loss and ice crystal size	(Payne and Young 1995)
Ocean pout	III	Chilled and frozen actomyosin	0.05 to 0.3 mg/L and 10 to 100 g/L	Freezing temperature depression and inhibition of ice recrystallization	(Boonsupthip and Lee 2003)

### Medical uses

#### Cryosurgery

Cryosurgery, commonly known as cryotherapy or cryoablation, is a procedure that uses freezing to remove unviable/unwanted tissues. Specifically, this therapy is becoming increasingly common as it has different medical benefits compared to conventional surgery, it is less intensive, less costly, and results in less suffering, bleeding, and other problems that often stem from the surgery.

Cryosurgery can still not be considered a routine method of cancer therapy. In certain clinical cases, freezing alone may only partially kill the targeted tumor, due to inadequate or improper freezing. In addition, healthy tissues around it may also suffer from freezing injury due to a large amount of cold emitted from the freezing probe (Bouvet and Ben 2003; Zhang et al. 2008). Several adjunctive therapies have been suggested to enhance the efficacy of cryosurgery, namely, chemical adjuvants such as cancer chemotherapeutic agents (Feeney and Yeh 1998; Kim et al. 2017; Raymond and DeVries 1972), AFP I (Bagis et al. 2008; Feeney and Yeh 1998; Leygonie et al. 2012; Rubinsky et al. 1994), amino acid adjuvants such as glycine (Wang et al. 2008) and tumor necrosis factor alpha (TNF-α) have all been investigated for this purpose (Goel et al. 2007).

The feasibility and efficacy of an amino acid glycine adjuvant was surveyed to improve the cryodestruction of MCF-7 human breast cancer cells under mild freezing/thawing circumstances via eutectic crystallization. The findings revealed that a NaCl–glycine–water mixture has two separate eutectic phase transition events that lead to binary eutectic water–glycine solidification, and NaCl–glycine–water ternary eutectic solidification (Wang et al. 2008).

AFPs damage RBCs at higher concentrations by causing the ice to form a needle-like shape, which can kill the cells (Carpenter and Hansen 1992). Cryosurgery is effective (Boonsupthip and Lee 2003; Chao et al. 1996; Modig et al. 2010) with Koushafar and Rubinsky (1997) suggesting that the use of type I AFP 10 mg/ml induces the degradation of human primary prostatic adenocarcinoma cells by intracellular ice formation. An in vivo model using subcutaneous tumor mice recommended that type I AFP injection into tumors prior to freezing could lead to higher devastation. The injury to tumors was evaluated through the measurement of its overall metabolic activity. The test yielded abnormal results when used to determine the severity of injury immediately after the treatment, underestimating the severity of the injury. However, a double-freeze method with protein anti-freezing available has been observed to provide substantially better ablation than a double-freeze without AFP or a single freeze with or without AFP (Muldrew et al. 2001; Pham et al. 1999).

#### Cryopreservation

In some medical viewpoints, two unique AFP properties, TH and IRI, may be of interest. The non-colligative freezing-point depression can be used to enhance hypothermic storage, while IRI plays a significant role in cryopreservation by securing membranes from freezing injury. There are currently a considerable amount of cryoprotectants (CPAs) that can be used to preserve cells and tissues. Most of the CPAs used, however, often demonstrate toxicity (Fuller 2004).

Model membrane research has shown that AFGPs prohibited the permeation of dielaidoylphosphatidylcholine (DEPC), dielaidoylphosphatidylethanolamine (DEPE) and dielaidoylphosphatidylglycerol (DEPG) (Ikekawa et al. 1985; Koushafar et al. 1997; Mir and Rubinsky 2002) and that AFGP7–8 provided minimal protection for the membranes of dimyristoylphosphatidylcholine (DMPC) with the insertion of differing quantities of galactolipids as it cooled through the phase transition temperature during cooling (Tomczak et al. 2001). Type I AFP has also been shown to interact with the acyl lipid chains in a mixture of DMPC and plant thylakoid lipid digalactosyldiacylglycerol (DGDG) (Tomczak et al. 2002) and to inhibit zwitterionic DEPC liposomes (Wu and Fletcher 2000). In some cases, AFGP1–5 and AFP type I were observed to induce concentration-dependent leakage from the liposomes and to be fusogenic to the liposomes (Wu and Fletcher 2000). Before cooling, AFPs didn't stop DEPG from being negatively charged or stop the leakage of DEPE liposomes as they were cooled through their transition temperature.

Currently, two methods are widely used in cryopreservation: gradual freezing (Rubinsky 2000) and verification (Fuller 2004). Nonetheless, after cryopreservation, many factors can affect the survival of cells and tissues, including freezing and warming levels, cell state and size, storage temperature, storage length, and CPA type and concentration.

Despite the fact that both TH and IRI activity of AFPs are derived from ice-binding behavior, they are not proportional, which means that hyperactive AFPs with high TH are not preferable to moderate AFPs with low TH in terms of IRI activity (Lee et al. 2015; Gruneberg et al. 2021). Ice recrystallization is a thermodynamically favorable process that results in the formation of large-sized ice crystals. This thawing phenomenon, which could also cause severe cryoinjury, is fatal to cells (Budke et al. 2006; Deller et al. 2015). The recombinant AFP had the same amino acid sequence as Type I AFP and was used as an additive of hydroxyethyl starch (HES) for cryopreservation of RBCs (Tomalty et al. 2019). The fact that hemolysis does not lessen monotonically with AFP density is an interesting finding. Cryomicroscopy shows that 1.54 mg/ml AFPs nearly completely inhibits ice recrystallization while increasing the damage to cryopreserved RBCs. A delicate balance between AFP-induced cell conserving and malfunction may exist, depending on IRI activity and preferable expansion of ice around the cells, respectively. Cryopreservation of ram spermatozoa was also noticed to be independent of AFPs concentration. While Type I AFP is toxic during the precooling phase, a concentration of 10 µg/ml AFP may substantially safeguard the spermatozoa (Robles et al. 2019). Higher concentrations of AFPs, on the other hand, would result in the creation of needle-shaped ice crystals, which can pierce the cell membrane and reduce the post-thaw survival of cryopreserved cells (Lee et al. 2020). In the year 2000, a conflicting characterization of AFPs in cryopreservation was proposed. As a result, the appropriate dose of AFPs is critical to cryopreservation, which is highly dependent on the type of AFPs and cells. Table 3 summarizes some reports on cryopreservation using AFPs.

**Table 3:** AFPs used for cryopreservation of biological samples

AFPs source	Type of AFPs	Biological sample	AFP Quantities	Function	References
Ocean pout (*Macrozoarces americanus*)	─	Rabbit parietal cells	0.1 to 20 mg/ml	Ca permeability	(Negulescu et al. 1992)
Fish	─	Equine embryos	20 mg/ml	Reducing cell damage	(Lagneaux et al. 1997)
Antarctic fishes	AFGP	Mouse embryos	40 mg/ml	Post thaw viability	(Rubinsky et al. 1992)
Fish	AFGP	Rat cardiomyocytes	0.5 to 10 mg/ml	Promote intracellular freezing	(Mugnano et al. 1995)
Winter flounder (*Pseudopleuronectes americanus*)	I	Human RBCs	5 to 160 µg/ml	Influence on ice crystal growth	(Carpenter and Hansen 1992)
Fish	I	Rat liver	1 mg/ml	Preventing ice formation	(Soltys et al. 2001)
Sub-arctic species winter flounder	I	Seabream (*Sparus aurata*) embryos	20 nL of 10 mg/ml	Improve chilling resistance	(Robles et al. 2007)
Winter flounder	I	Rat hippocampal slice cultures	10 mg/ml	Protects neurons from hypothermia/re-warming injury	(Rubinsky et al. 2010)
North Atlantic notothenioid fish (*Marcrozoarces americanus*)	III	Buffalo bull sperm	0.01, 0.1, 1, and 10 mg/ml	Improving the progressive motility and plasma membrane integrity	(Qadeer et al. 2014)
Notched-fin eelpout (*Zoarces elongatus Kner*)	III	Human HepG2	2 to 10 mg/ml	Improving cell viability	(Hirano et al. 2008)
Ocean pout (*Macrozoarces americanus*)	III	Chimpanzee (pan troglodytes) spermatozoa	1, 10, and 100 µg/ml	Improves post-thaw sperm motility	(Younis and Gould 1998)
Winter flounder	I	Bovine oocyte	20 mg/ml	Reducing the leakiness of the cell membranes	(Rubinsky et al. 1991)
Sea raven	II				
Ocean pout	III				
Diatom (*Navicula glaciei)*	NgIBP	Human RBCs	25, 50, and 77 µg/ml	Reducing cell damage	(Kang and Raymond 2004)
Winter flounder (*Pseudopleuronectes americanus*)	I	Human RBCs	0 to 1.54 mg/ml	Inhibition of ice recrystallization	(Chao et al. 1996)
Sea raven (*Hemitripterus americanus*)	II				
Antarctic and Arctic fishes	AFGP	Pig oocytes	0.1, 1, and 40 mg/ml	Protection of membranes at hypothermic temperatures	(Rubinsky et al. 1990)
Winter flounder	I	Sheep embryo	1 or 10 mg/ml	Enhancing store time	(Baguisi et al. 1997)
Ocean pout	III				
*Liopsetta pinnifasciata*	I	Rat insulin cells	10 mg/ml	Prolonging cell lifetime	(Kamijima et al. 2013)
*Hypomesus japonicus*	II				
*Zoarces elongates Kner*	III				
*Eleginus gracilis*	AFGP				

### Other applications

These proteins can be used as green de-icing agents. To date, researchers funded by the national science foundation (NSF) have successfully introduced two of the four different types of fish AFPs into yeast and bacteria by recombinant DNA technology. The vitrification process is another potential application for AFPs. At present, vitrification requires high cooling rates and high concentrations of various, more or less toxic, low molecular weight substances. AFPs can be used in the future to stabilize super-cooled fluids until the solution vitrifies. This could reduce problems with low volume and high concentrations of low molecular weight substances.

Inhibiting the growth of ice in this condition by stabilizing solutions with AFPs can also become a possibility. However, since the super-cooled condition is metastable, maintaining this condition can contribute to high concentrations of AFPs. Therefore, it can be envisaged that only costly products will be stored using this method in the near future. These products may be more pertinent to the medical industry than to the food processing industry, as one may envision the organ banks in which the organs are kept super-cooled instead of frozen (Ramløv and Johnsen 2014).

## Benefits and problems of using AFPs

However, AFPs have been known for about 40 years, the production of AFPs in quantity and at a low production cost has only become technically feasible recently. AFPs' recrystallization-inhibiting effect is already being operated in different food products, improving both their nutritive value when kept in cold storage. The introduction of AFPs as a food maintainer will boost the reliability of frozen foods and desserts during storage, shipping, and thawing. One of the strengths of AFPs for the food industry is that they could be relatively active at such low rates that these food ingredients may become quite economical. Although our understanding of ISPs is not so advanced to establish the exact mechanisms of action, it has reached the level of knowledge of basic structures to be able to invent new AFPs or ISPs. More investigations are necessary to check the potential of special processing (e.g., enzymatic reaction) on the protection and nutritional ability of possible products for application to AFPs.

Fish AFPs have one specific feature as a possible food additive, which can cause problems. Ice grown from a solution having fish AFP will take the form of spicules or needles (Grandum et al. 1999; Wilson et al. 2002); crystals shaped in these solutions may make the product less pleasant. The same issue does not apply to insect AFPs, since the ice crystals produced in such solutions are much rounder, not specular, or needle-like. AFPs are operational at temperatures below freezing. This could indicate that there is little optional pressure on thermally constant proteins and, consequently, most AFPs are not only fundamentally solid but also flexible (Marshall et al. 2005); that assumption could prove troublesome for future applications in the food industry (Garcia-Arribas et al. 2006). In food production, any inserted AFP may also require to endure heat treatment (pasteurization), although specific AFPs tend to have low denaturation temperature (Cheng and DeVries 1989; Li et al. 1991). This leads to unintentional decadence or irreversible association and consequently to inactivation during storage and/or ultimately to heat treatment, such as pasteurization (Wang et al. 2002). There are still several concerns that need to be fixed, such as cytotoxicity, low abundance in nature and stability, before AFGPs can find universal usage. Though initial results of AFP types I and III indicate that these compounds do not cause long-term health effects or immunogenicity (Crevel et al. 2002; Hall-Manning et al. 2004; Pham et al. 1999). There was only one AFGP cytotoxicity study (Hincha et al. 1993), but that was not in a mammalian environment. Liu et al. (2007) have shown that adding high doses of AFGP8 to human liver and kidney cells rises caspase-3/7 activity, resulting in cell death through an apoptotic process. This cytotoxic impact is possibly due to an innate activation of apoptosis, as AFGP8 is swiftly internalized by human liver cells, possibly through endocytosis based on ATP. Despite AFGP8's many useful anti-freezing characteristics, cytotoxicity in vitro would have a drastic effect on the potential use of native AFGP8 for cryoprotection and hypothermic storage.

## Conclusions

The unique properties of AFPs as biological anti-freezing agents have attracted interest from researchers in academia and particularly in the biomedical fields. Over the past 50 years, studies have revealed on a number of specific AFPs that seemed to have diverse structures and functions. In this review, we surveyed the property of freezing point depression, termed TH, the ability to inhibit IRI and AFPs' function to affect the DIS and its interaction with membranes. AFPs have received significant attention concerning freeze resistance and avoidance in organisms, food processing, cryopreservation, and ice slurries.

There may be some commercial applications for such AFPs. These compounds at the same concentrations are about 300 times more effective in preventing freezing than conventional chemical anti-freezing agents. The efficiency of AFPs in inhibiting ice growth suggests that they could be used in several applications to prevent food freezing and freezing injury. For example, they could be used for cryopreservation of foods that are normally made inedible due to ice crystals damage or to increase the cold resistance of living plants, as well as allow for cryopreservation of tissues and organs. AFPs used for food preservation could improve desserts and frozen food storage time and thawing quality. Moreover, the fact that they are very active at low concentrations could ease the overall cost-effectiveness of the products.

AFPs are becoming more common in marine species. Many of the marine AFPs recently identified are more hyperactive than those from fish. This hyperactivity is considered to be useful particularly for transgenic technologies and hypothermic storage. Protein engineering, synthetic biology, and structural biology all contribute to tailoring existing AFPs to make them suitable for medical and other commercial applications.

## Data Availability

Not applicable.

## Abbreviations

AFPs: Antifreeze proteins
TH: Thermal hysteresis
IRI: Ice recrystallization inhibition
AFGPs: Antifreeze glycol proteins/peptides
ISPs: Ice structuring proteins
HFP: Hysteresis freezing point
NGS: Next-generation sequencing
IIF: Intracellular ice forming
RBC: Red blood cell
NMR: Nuclear magnetic resonance
CTLDs: Lectin-like domains
SAS: Sialic acid synthase
QAE: Quaternary-amino-ethyl
SP: Sulfopropyl
LHS: Longhorn sculpin plasma
CD: Circular dichroism
Apos: Apolipoproteins
TLP: Trypsino-gen-like protease
IBP: Ice binding proteins
HGT: Horizontal gene transfer
MP: Melting point
FWIP: Falling water ice affinity purification
CPAs: Cryoprotectants
DEPC: Dielaidoylphosphatidylcholine
DEPE: Dielaidoylphosphati-dylethanolamine
DEPG: Dielaidoylphosphatidylglycerol
DMPC: Dimyristoylphosphatidylcholine
NSF: National science foundation
SP: Sulfopropyl
DGDG: Digalactosyldiacylglycerol
IBS: Ice-binding site
PAMAM: Polyamidoamine
HES: Hydroxyethyl starch
PPII: Polyproline type II
DIS: Dynamic ice shaping
TNF-α: Tumor necrosis factor alpha
